# An Interpretable Deep Learning Model for Covid-19 Detection With Chest *X*-Ray Images

**DOI:** 10.1109/ACCESS.2021.3087583

**Published:** 2021-06-08

**Authors:** Gurmail Singh, Kin-Choong Yow

**Affiliations:** Faculty of Engineering and Applied SciencesUniversity of Regina 6846 Regina SK S4S 0A2 Canada

**Keywords:** Covid-19, pneumonia, image recognition, *X*-ray, prototypical part, deep learning

## Abstract

Timely and accurate detection of an epidemic/pandemic is always desired to prevent its spread. For the detection of any disease, there can be more than one approach including deep learning models. However, transparency/interpretability of the reasoning process of a deep learning model related to health science is a necessity. Thus, we introduce an interpretable deep learning model: Gen-ProtoPNet. Gen-ProtoPNet is closely related to two interpretable deep learning models: ProtoPNet and NP-ProtoPNet The latter two models use prototypes of spacial dimension $1\times 1$ and the distance function $L2$. In our model, we use a generalized version of the distance function $L2$ that enables us to use prototypes of any type of spacial dimensions, that is, square spacial dimensions and rectangular spacial dimensions to classify an input image. The accuracy and precision that our model receives is on par with the best performing non-interpretable deep learning models when we tested the models on the dataset of $X$-ray images. Our model attains the highest accuracy of 87.27% on classification of three classes of images, that is close to the accuracy of 88.42% attained by a non-interpretable model on the classification of the given dataset.

## I. Introduction

The world is still struggling with the pandemic of Covid-19 and its variants, such as: B.1.1.7, B.1.351 and P.1 [18]. Many facets efforts have been made to control and contain the disease. These efforts include detection of the virus. Many models have been proposed to detect Covid-19 from medical images, see [4], [12], [22], [23], [25], [36], [37], [40], [58]. These models lack the interpretability of their predictions, but the interpretability of the models related to public health is utmost important. The objective of this work is to find an interpretable method to do image classification so that we can tell why an image is classified in a certain way. In this work, we introduce an interpretable deep learning model: *generalized prototypical part network* (Gen-ProtoPNet), and experiment it over the dataset of three different classes of $X$-rays, see Section V. Gen-ProtoPNet is a close variation of ProtoPNet [7] and NP-ProtoPNet [41]. To predict the class of a test image, ProtoPNet calculates the similarity scores between learned prototypical parts (with square spacial dimensions $1 \times 1$) of images from each class and parts of the test image using $L2$ distance function. These similarity scores are multiplied with a weight matrix to establish a positive connection between prototypes and logits of their correct classes, and a zero connection between prototypes and logits of incorrect classes. However, NP-ProtoPNet establishes a negative connection between prototypes and logits of incorrect classes instead of a zero connection between prototypes and logits of incorrect classes, unlike ProtoPnet. Both ProtoPNet and NP-ProtoPNet use the distance function $L2$ to calculate similarity scores, and they use prototypes of square spacial dimensions $1\times 1$. In this work, we use a generalized version of the distance function $L2$, see Section V-A. The generalized version of the distance function enables us to use prototypical parts of any type of spacial dimensions, that is, rectangular spacial dimensions as well as square spacial dimensions. In this work, a prototypical part or a prototype will represent a part, a patch or a section of an image. The similarity score between a learned prototypical part and an image is considered better if the sum of squares of the differences between the corresponding pixel values of the prototype and a patch of the image is lesser. We are motivated not to use $1\times 1$ spacial dimensions for prototypes due to following two reasons: (i) the model gives lesser accuracy; (ii) higher accuracy with the wrong reasoning process.

First, small spacial dimensions ( $1\times 1$) can lead to lesser accuracy because images of objects from different classes can have small parts similar that can lead to good similarity score between patches of a test image and patches of images from wrong classes that gives rise to wrong classification. For example, most of the $X$-ray images have some part black as a background, see Figure 2. Therefore, the use of prototypical parts of spacial dimensions $1\times 1$ can give good similarity scores between patches of an input image and patches of images from wrong classes, because all the images have some part black. Another example, images of birds from different sea bird species can share same background on most part of the images. So, the use of prototypes of spacial dimension $1\times 1$ can wrongly give high similarity score between patches of a test image and patches of images form wrong classes, because mostly such images have water as a background.

**FIGURE 1.**
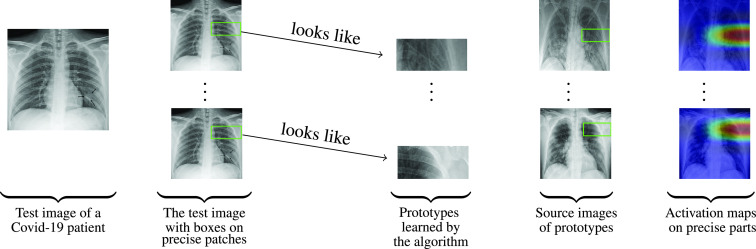
Similarity between learned prototypical parts and an $X$-ray image of a Covid-19 patient.

**FIGURE 2.**
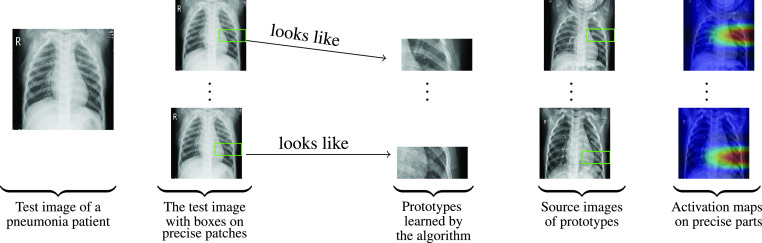
Similarity between learned prototypical parts and an $X$-ray image of a pneumonia patient.

Second, the images of objects from completely different classes do not have even small patches similar, and the classification of the images can be made just on the basis of the background in the images instead of identifying the objects themselves. For example, any patch of the images of birds of a sea specie is not similar to any patch of the images of birds of a jungle specie. Therefore, the use of the prototypical parts of the spacial dimensions $1\times 1$ can separate a sea bird image from an image of a jungle bird just on the basis of the background in the images instead of identifying the birds themselves.

On the other hand, using prototypical parts of spacial dimension of the biggest possible size can also reduce the accuracy because then the prototypical part will be an image itself instead of being a part of an image. Therefore, there can be only few images that are very similar to such a prototype whose size is equal to the size of an image, but a part of an image with smaller size can be similar to the parts of many other images. Since a prototype represents a part of an image, the optimum value for the spacial dimensions of a prototype lies between $1\times 1$ and the biggest possible dimension (in our case, the biggest possible spacial dimension of prototypes is $7 \times 7$), that is, the spacial dimension of the output of the convolution layers of the baseline models, see Section V.

The use of prototypical parts of spacial dimension bigger than $1\times 1$ gives us better accuracy and precision as compared to prototypical parts of spacial dimensions $1\times 1$ that are used in ProtoPNet and NP-ProtoPNet, see Section VIII.

## II. Literature Review

Some techniques have been developed to interpret the convolution neural networks, such as: posthoc interpretability analysis and attention-based interpretability. In *posthoc* analysis of a deep learning model, one interprets a trained convolution neural network after the predictions with the fitting explanations of the reasoning behind classifications made by the model. Posthoc analysis is made with various techniques, such as: activation maximization [10], [19], [27], [35], [43], [48], [57], deconvolution [59], and saliency visualization [39], [43], [45], [46]. However, these posthoc visualization methods fail to explain the actual reasoning process behind the classifications made by the models.

Many models have been developed that build attention-based interpretability, such as: class activation maps and part-based models. An attention-based interpretability model attempts to highlight the parts of an input image on which the network focuses [11], [15], [16], [21], [38], [42], [47], [56], [60]–[63]. Nevertheless, these models have serious drawback of not pointing out the prototypical parts that are similar to patches of the image on which the model focuses.

Li *et al.* [28], proposed a deep learning architecture that builds case-based reasoning into a neural network. Then Chen *et al.* [7] along with the authors of the above paper made a considerable improvement in their model ProtoPNet, whereby the network makes prediction by comparing image patches with the learned prototypes. The authors of this paper introduced a model NP-ProtoPNet [41] that is a close variation of ProtoPNet.

As mentioned in the introduction, many networks have been emerged to classify the $X$-ray images of Covid-19 patients along with $X$-ray images of normal people and pneumonia patients, see [4], [12], [22], [23], [25], [36], [37], [40], [41], [58]. A study summarizes some papers on Covid-19, and it points out some problems, such as: lack of reliable and adequate amount of data for deep learning algorithms [5]. Some studies related to Covid-19 and IoT are also worth mentioning [1]–[3], [6], [8], [26], [30]–[33], [49]. In this work, we also experimented our model over the same dataset that is used in some of the above Covid-19 related articles.

## III. Data

We trained and evaluated our network on the dataset of frontal chest $X$-ray images of Covid-19 patients [13], pneumonia patients and normal persons [24]. The dataset of chest $X$-ray images from Kaggle database [24] has 3875 and 1341 training images of pneumonia patients and normal persons, respectively. Also, the dataset has 390 and 234 test images of pneumonia patients and normal persons, respectively. The other database [13] has 930 medical images of Covid-19 patients. These medical images include, frontal chest $X$-ray images, CT-scan images, side $X$-ray images and few obscure (completely black or white) images. Among the medical images of Covid-19 patients, we selected only 748 frontal chest $X$-ray images. As compared to the number of chest $X$-ray images of pneumonia patients and normal persons, the number of chest $X$-ray images of Covid-19 patients was much lesser. Therefore, to balance the data, a copy of the chest $X$-ray images of Covid-19 patients was included to form a dataset of 1496 images. The 1496 frontal chest $X$-ray images of Covid-19 patients were divided into train and test sets of 1248 and 248 images, respectively. All these images form the three classes, labeled as: Covid, Normal and Pneumonia. We resized the images to dimension $224\times 224$.

## IV. Working Principle and Novelty of Gen-ProtoPNet

For $X$-ray images given in figures 1 and 2, ProtoPNet is able to recognize many patches of the test image that looks like parts of images of some class. ProtoPNet decides the class of a test image on the basis of a weighted combination of the similarity scores [7]. Similarity scores between patches of a test image and prototypical parts (with square spacial dimensions $1\times 1$) are acquired with maximization of the inverted $L2$ distances between them. The novelty of our model Gen-ProtoPNet is as follows.

1) Our model uses a generalized version (see Section V-A) of the distance function $L2$.
2) It uses prototypes of both types of spacial dimensions, that is, it uses prototypes of square spacial dimensions as well as rectangular spacial dimensions.
3) It uses prototypes with spacial dimensions bigger than the square spacial dimensions $1\times 1$, that is, either height or width of prototypes is bigger than 1.

The use of generalized distance function and spacial dimensions bigger than $1\times 1$ helped our model to improve its performance.

## V. Methodology

**FIGURE 3.**
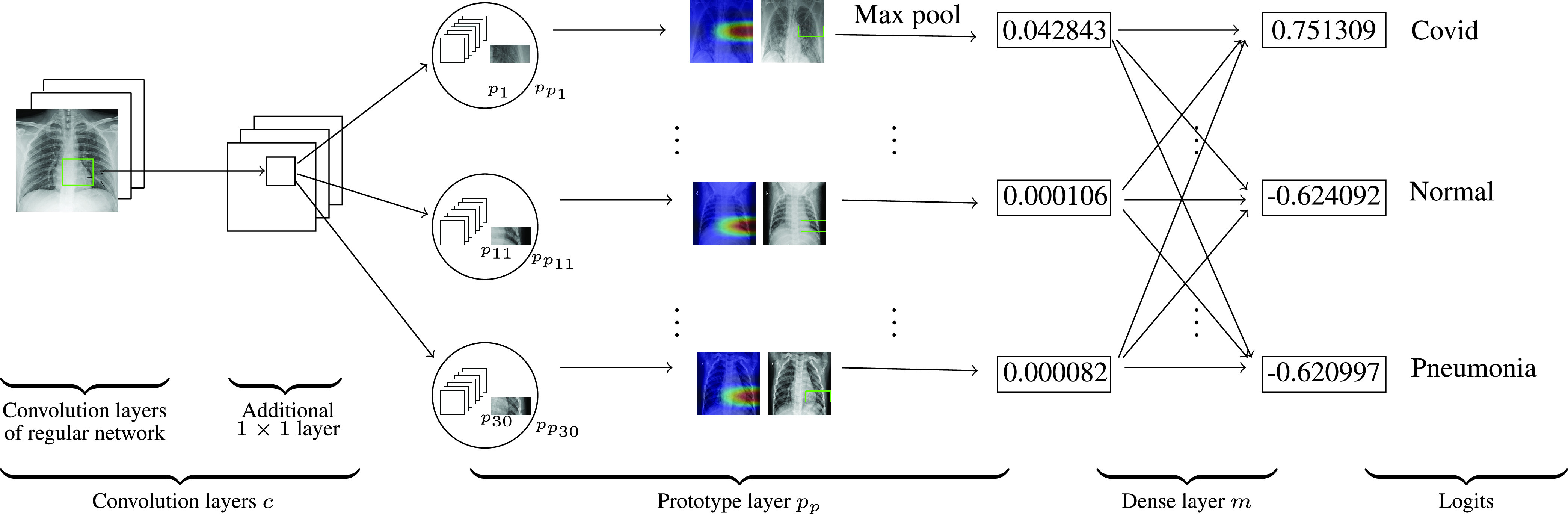
Gen-ProtoPNet architecture.

We construct our model over the following models: VGG-16, VGG-19 [44], ResNet-34, ResNet-152 [17], DenseNet-121, or DenseNet-161 [20] (initialized with filters pretrained on ImageNet [9]). We call these models baseline or base models. In the Figure 3, we see that the model comprises of the convolution layers of any of the above base model that are followed by an additional $1\times 1$ layer (we denote these convolution layers together by $c$ whose parameters are collectively denoted by $c_{conv}$) and then these convolution layers are followed by a prototype layer $p_{p}$ and a fully connected layer $m$ with weight matrix $wt_{m}$ and no bias. The prototype layer $p_{p}$ is a generalized convolution layer, [14], [34]. The activation function ReLU is used for all the convolution layers. Note that, Gen-ProtoPNet has only one additional $1\times 1$ convolution layer unlike ProtoPNet.

Let $x$ be an input image and $c(x)$ is the output of the convolutional layers $c$. If $D$ is the depth of the output $c(x)$ then the shape of $c(x)$ is $D\times 7 \times 7$. For example, when Gen-ProtoPNet is constructed over the convolution layers of VGG-16 then the depth of $c(x)$ is 512. Although, there is one additional $1 \times 1$ convolution layer, but it is not used to reduce the depth of $c(x)$. Therefore, the depth of each prototype is set equal to the depth of output of the regular convolution layers. We pick 10 prototypical parts for each class of images, and this number is randomly chosen. That is, Gen-ProtoPNet learns 30 prototypes. The set of prototypes is denoted by $P = \{p_{r}\}^{30}_{r=1}$. These prototypical parts should catch enough relevant parts for recognizing a test image. The shape of each prototype is $D\times h \times w$, where $1 \times 1 < h \times w < 7 \times 7$, that is, both $h$ and $w$ are not simultaneously equal to 1. The spacial dimensions of $c(x)$ are $7 \times 7$, whereas the spacial dimensions of the prototypes are $h\times w$. Thus, every prototypical part $p_{r}$ will be used to represent some prototypical activation pattern in a patch of $c(x)$. Hence, every prototypical part $p_{r}$ can be considered as a representation of a patch of some image.

In Figure 3, the prototypical part $p_{1}$ is similar to a part of the $X$-ray of a Covid-19 patient. The original/source image of the prototypical part $p_{1}$ is also given in the Figure 3. The patch $p_{1}$ is the part of the original image, that is enclosed in a rectangle with green boundaries. Similarly, the patches $p_{11}$ and $p_{30}$ are parts of the original images given in the same row in the Figure 3. For the output $z = c(x)$ of a test image $x$, the $r$-th prototypical unit $p_{p_{r}}$ in $p_{p}$ calculates (with the generalized version of $L2$) distances between the prototypical part $p_{r}$ and each patch of $z$. These distances are inverted into similarity scores which results in an activation map of similarity scores. More the activation value, stronger the presence of a prototype in the image $x$. This activation map preserves the spatial relation of the convolutional output, and can be upsampled to the size of the input image to produce a heat map that identifies which part of the input image is most similar to the learned prototype [7]. These regions are enclosed in the green rectangles on the source images. The activation map is max-pooled to reduce to a single similarity score, that is, there is only one similarity score for each prototype. In the fully connected layer $m$, the similarity scores produced with global max-pooling are multiplied with the matrix $wt_{m}$ to get the logits, then these logits give prediction after normalization with softmax.

The $(r, s)$-th entry $wt^{(r,s)}_{m}$ of the weight matrix $wt_{m}$ connects $s$-th prototype and the logit of $r$-th class. The similarity scores (after max-pooling) form a column matrix $S$, see Section VI. The logits of Covid, Normal and Pneumoina classes are obtained from the multiplication of first, second and third rows of $wt_{m}$ with the matrix $S$. In Figure 3, the test image is an $X$-ray image of a Covid-19 patient. The prototypes $p_{1}-p_{10}$, $p_{11}-p_{20}$ and $p_{21}-p_{30}$ are prototypes of images from Covid, Normal and Pneumonia classes, respectively. The similarity scores between patches of the input image and patches $p_{1}$, $p_{11}$ and $p_{30}$ are 0.042843, 0.000106 and 0.000082, respectively. The complete list of similarity scores is provided in the similarity score matrix $S$, see Section VI. The logits for the classes Covid, Normal and Pneumonia are 0.751309, −0.624092 and −0.620997, respectively.

### A. Mathematical Formulation and the Training of Gen-ProtoPNet

In this section, we describe the generalization of the distance function $L2$ (Euclidean distance) using base model VGG-16. Also, we present the mathematical formulation and training steps of our algorithm with the generalized distance function. Gen-ProtoPNet is constructed over the regular convolution layers whose output channels have spacial dimension $7 \times 7$, see Section V. Let $x$ be an input image. Let $z$ ( $= c(x)$) be of the shape ( $D$, 7, 7), where $D$ is the depth of $c(x)$. Consider a prototype $p$ of the shape ( $D$, $h$, $w$). Let $z_{ijk}$ and $p_{lmk}$ be $(i,j)$ and $(l,m)$ pixels of $k$th tensor of $z$ and $p$, respectively. Let $zp$ be obtained by convolving $p$ over $z$ with stride size equal to 1. Then $zp$ is a tensor of the shape ( $D$, $8-h$, $8-w$). Therefore, each feature map of $zp$ has ( $8-h$)( $8-w$) pixels. For $0\leq i \leq 7-h$, $0\leq j \leq 7-w$ and $0\leq k \leq D-1$; $(i,j)$ pixel $(zp)_{ijk}$ of the $k$th feature map of $zp$ is given by:

\begin{align*}&\hspace {-1pc} z_{ijk}p_{00k}+\cdots +z_{i(j+w-1)k} p_{0(w-1)k} \\&+\, z_{(i+1)jk}p_{10k}+\cdots +z_{(i+1)(j+w-1)k} p_{1(w-1)k} \\&+ \qquad \qquad \qquad \cdots \\&+\, z_{(i+h-1)jk}p_{(h-1)k}+\cdots +z_{(i+h-1)(j+w-1)k} p_{(h-1)(w-1)k}. \\\tag{1}\end{align*}

Let $z^{2}$ be obtained from the Hadamard multiplication of feature maps of $z$ with themselves. Let $\mathcal {Z}^{2}$ be obtained from $z^{2}$ by convolving (over $z^{2}$) all 1’s filter of the shape of prototypes with stride size equal to 1. Note that, $\mathcal {Z}^{2}$ is the sum of the patches of $z^{2}$ of the shape $h\times w$ over all feature maps. Therefore, the shape of $\mathcal {Z}^{2}$ is $(8-h)\times (8-w)$, and $(i,j)$ pixel $\mathcal {Z}^{2}_{ij}$ of $\mathcal {Z}^{2}$ is given by:

\begin{align*}&\hspace {-1pc}\sum \limits ^{D-1}_{k=0} \big (z^{2}_{ijk}+z^{2}_{i(j+1)k}+\cdots +z^{2}_{i(j+w-1)k} \\&+\, z^{2}_{(i+1)jk}+z^{2}_{(i+1)(j+1)k}+\cdots +z^{2}_{(i+1)(j+w-1)k} \\&+\, \qquad \qquad \qquad \cdots \\&+\, z^{2}_{(i+h-1)jk} +z^{2}_{(i+h-1)(j+1)k}+\cdots +z^{2}_{(i+h-1)(j+w-1)k}\big). \\\tag{2}\end{align*}

Note that, $z$ has $(8-h)(8-w)$ patches of the spacial dimension $h\times w$. Hence, the distance $d^{2}(\mathcal {Z}_{ij}, p)$ between $(i,j)$ patch $\mathcal {Z}_{ij}$ (say) of $z$ and a prototype $p$ is given by:

\begin{equation*} d^{2}(\mathcal {Z}_{ij}, p)= \mathcal {Z}^{2}_{ij}-2\sum \limits ^{D-1}_{k=0}(zp)_{ijk} + \sum \limits ^{D-1}_{k=0}\sum \limits ^{h}_{l=1}\sum \limits ^{w}_{m=1} p^{2}_{lmk}.\tag{3}\end{equation*}

The equations 1 and 2 give the values of $(zp)_{ijk}$ and $\mathcal {Z}^{2}_{ij}$. Thus, $(zp)_{ijk} = \sum \limits ^{h-1}_{l=0}\sum \limits ^{w-1}_{m=0}z_{(i+l)(j+m)k}p_{(1+l)(1+m)k}$ and $\mathcal {Z}^{2}_{ij} = \sum \limits ^{D-1}_{k=0} \sum \limits ^{h-1}_{l=0}\sum \limits ^{w-1}_{m=0}z^{2}_{(i+l)(j+m)k}$. Therefore, by equation 3,

\begin{equation*} d^{2}(\mathcal {Z}_{ij}, p)= \sum \limits ^{h-1}_{l=0}\sum \limits ^{w-1}_{m=0}\sum \limits ^{D-1}_{k=0}||z_{(i+l)(j+m)k}-p_{(1+l)(1+m)k}||^{2}_{2}.\end{equation*}

If the spacial dimension of a prototype $p$ is $1\times 1$ then $h=w=1$ and $d^{2}(\mathcal {Z}_{ij}, p)= \sum \limits ^{D-1}_{k=0} ||z_{ijk}-p_{11k}||^{2}_{2}$, which is the square of $L2$ distance between a patch of $z$ and the prototype $p$, where $p_{11k}\simeq p_{k}$. Therefore, if the spacial dimensions of $p$ are not equal to $1\times 1$ then $d^{2}$ is a generalization of the distance function $L2$. The distance function $L2$ is used in both ProtoPNet and NP-ProtoPNet to find distances of prototypes (spacial dimension $1\times 1$) and the patches of images. The prototypical unit $p_{p}$ calculates:

\begin{equation*} p_{p}(z) = \max _{0\leq i \leq 7-h, 0\leq j \leq 7-w} \log \left ({\dfrac {d^{2}(\mathcal {Z}_{ij}, p)+1}{d^{2}(\mathcal {Z}_{ij}, p)+\epsilon }}\right).\end{equation*}

Alternatively,

\begin{equation*} p_{p}(z) = \max _{\mathcal {Z}\in {~\text {patches}}(z)} \log \left ({\dfrac {d^{2}(\mathcal {Z}, p)+1}{d^{2}(\mathcal {Z}, p)+\epsilon }}\right).\tag{4}\end{equation*}

The equation 4 tells us that if $\mathcal {Z}$ is similar to $p$ then $d^{2}(\mathcal {Z}, p)$ is smaller. The following three steps are performed to train our algorithm.

#### 1) Stochastic Gradient Descent (SGD) of Every Layer Before Dense Layer

At this stage of learning, Gen-ProtoPNet aim to learns important features of the image while salient parts cluster near their respective classes. To attain this aim, Gen-ProtoPNet collectively optimize the parameters $c_{conv}$ and $p_{1}-p_{30}$ in $p_{p}$ using SGD. Let $X= \{x_{1}\cdots x_{n}\}$ be a set of image and $Y= \{y_{1}\cdots y_{n}\}$ is a set of corresponding labels, and $D = \{(x_{r}, y_{r}): x_{r}\in X, y_{r} \in Y\}$. Our goal is to solve the following optimization problem:

\begin{align*}&\hspace {-0.5pc}\min _{P, c_{conv}} \dfrac {1}n\sum \limits ^{n}_{r=1}\text {CrsEnt}(h\circ p_{p}\circ c(x_{r}), y_{r})+\lambda _{1}\text {ClstCt} \\&\qquad \qquad \qquad \qquad \qquad \qquad \qquad \qquad +\,\lambda _{2}\text {SepCt},\tag{5}\end{align*}

where ClstCt and SepCt are as follows:

\begin{align*} \text {ClstCt}=&\dfrac {1}{n}\sum \limits ^{n}_{r=1}\min _{s: p_{s}\in P_{y_{r}}}\min _{\mathcal {Z}\in \text {patches}(c(x_{r}))}d^{2}(\mathcal {Z}, p_{s}); \tag{6}\\ \text {SepCt}=&- \dfrac {1}{n}\sum \limits ^{n}_{r=1}\min _{s: p_{s}\not \in P_{y_{r}}}\min _{\mathcal {Z}\in \text {patches}(c(x_{r}))}d^{2}(\mathcal {Z}, p_{s}).\tag{7}\end{align*}

The decrease in cluster cost clusters prototypical parts around their correct class, see equation 6, whereas the decrease in separation cost attempts to separate prototypical parts from their incorrect class [7], see equation 7. The decrease in the cross entropy gives better classifications, see equation 5. The coefficients $\lambda _{1}$ is set equal to 0.8 and the coefficient $\lambda _{2}$ belongs to the interval (0.08, 0.8). Let $P_{r}$ be the set of prototypical parts of the images that belong to $r$-th class. For a class $r$, we put $wt^{(r,s)}_{m}=1$ for all $s$ with $p_{s} \in P_{r}$ and $wt^{(r,s)}_{m}=-0.5$ for all $s$ with $p_{s} \not \in P_{r}$. Since similarity scores are nonnegative, in this way Gen-ProtoPNet learns a meaningful latent space [7].

#### 2) Push of Prototypical Parts

To see which part of the training images are used as prototypes, Gen-ProtoPNet projects every prototype $p_{s}$ onto the patch of the output $c(x)$ that has smallest distance from $p_{s}$, and $x$ belong to class of $p_{s}$ [7]. That is, for every prototype $p_{s}$ of class $r$, Gen-ProtoPNet perform the following update:

\begin{equation*} p_{s} \longleftarrow \min _{ \{\mathcal {Z}: \mathcal {Z} \in \text {patches}(c(x_{k})) \forall k {~\text {s.t. }} y_{k} = r\}}d^{2}(\mathcal {Z}, p_{s}).\end{equation*}

#### 3) Optimization of the Last Layer

To rely only on positive connections between prototypes and logits. We aim to make negative connection $wt^{(r,s)}_{m}$ to 0 for all $s$ with $p_{s} \not \in P_{r}$. We perform this process after fixing all the parameters of convolution layers and prototype layer, and aim to optimize [7]:

\begin{equation*} \min _{P, c_{conv}}\dfrac {1}n\sum \limits ^{n}_{k=1}\text {CrsEnt}(h\circ p_{p}\circ c(x_{k}), y_{k})\!+\!\lambda \sum \limits ^{3}_{r=1}\sum _{s: p_{s}\not \in P_{r}}|wt^{(r,s)}_{m}|.\end{equation*}

### B. Selection of an Image Patch as a Prototype

Suppose $x$ is the source image of a prototype $p_{r}$. The patch of $x$ that is most activated by the prototype $p_{r}$ is used for the visualization of $p_{r}$. Its activation value must be at least 92nd percentile of all the activation values (before max-pooling) of $p_{p_{r}}$ [7].

## VI. Explanation of the Reasoning Process of Gen-ProtoPNet With an Example

We constructed our model over six baseline models. We trained and tested our model for 500 epochs. The model VGG-16 is used as a baseline model to run the experiments explained in this example. However, the measures of the performance of the model with the other baseline model are given in the Table 1.

**TABLE 1:** Comparison of the Performances of Gen-ProtoPNet and the Other Models

Base (B)	Dimension/Metrics	Gen-ProtoPNet+B	NP-ProtoPNet[27] + B	ProtoPNet[2] + B	B only
VGG-16	$4 \times 6$				
	accuracy	85.89	84.63	79.73	82.45
	precision	0.99	0.97	0.87	0.97
	recall	0.98	0.99	0.92	0.98
	Fl-score	0.98	0.97	0.89	0.97
VGG-19	$3 \times 6$				
	accuracy	84.40	88.99	81.78	82.68
	precision	0.99	0.91	0.84	0.96
	recall	0.98	0.96	0.95	0.98
	Fl-score	0.98	0.93	0.89	0.97
ResNet-34	$3 \times 3$				
	accuracy	85.20 ± 0.23	84.29 ± 0.10	82.90 ± 0.21	84.89 ± 0.12
	precision	0.99	0.99	0.98	0.99
	recall	0.98	0.99	0.99	0.99
	Fl-score	0.98	0.99	0.98	0.99
ResNet-152	$4 \times 6$				
	accuracy	86.70 ± 0.18	86.40 ± 0.12	83.60 ± 0.34	88.03 ± 0.24
	precision	0.98	0.99	0.87	0.98
	recall	0.99	0.99	0.89	0.99
	Fl-score	0.98	0.99	0.88	0.98
DenseNet-121	$3 \times 5$				
	accuracy	87.04 ± 0.13	86.47 ± 0.11	85.90 ± 0.36	88.41 ± 0.12
	precision	0.98	0.99	0.98	0.99
	recall	0.99	0.98	0.98	0.99
	Fl-score	0.98	0.98	0.98	0.99
DenseNet-161	$3 \times 3$				
	accuracy	87.27 ± 0.23	87.04 ± 0.21	86.58 ± 0.32	88.42 ± 0.23
	precision	0.97	0.97	0.95	0.99
	recall	0.99	0.99	0.96	0.99
	Fl-score	0.97	0.97	0.96	0.99

**FIGURE 4.**
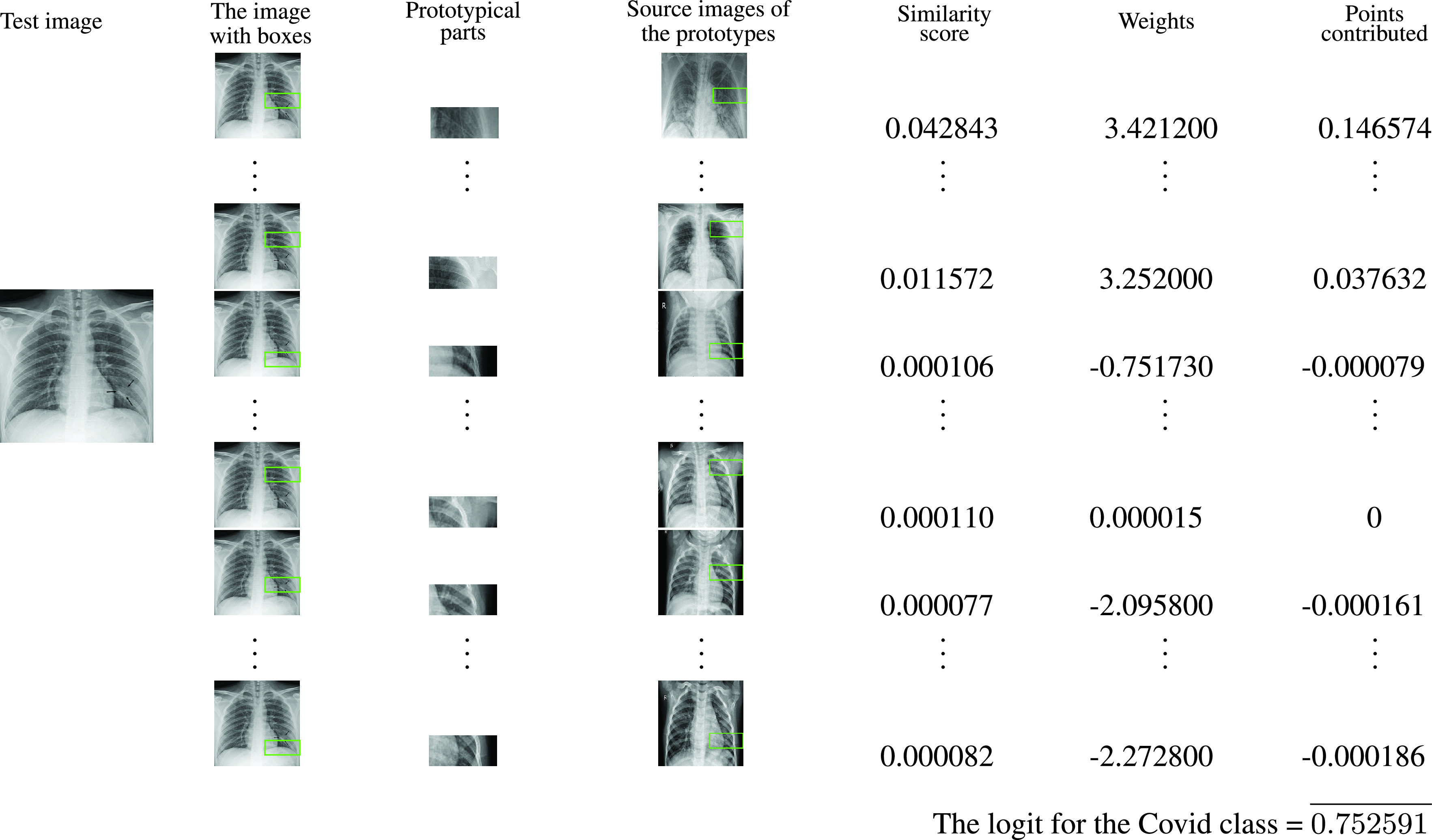
The explanation of the reasoning process of our network with an $X$-ray image of a Covid-19 patient.

In the Figure 4, the test image in the first column is a member of the Covid class. In next column, each image is the test image with a rectangle (at a certain place) on it. The rectangles have green boundaries. The pixels enclosed by such a rectangle on an image in the second column correspond to the pixels on the original image in the fourth column and same row. In fact the patch enclosed by a rectangle on an image in the fourth column is the patch from where a prototype (third column and same row) is projected. The fifth column consists of similarity scores that are explained in Section V. The similarity scores of a prototype $p_{s}$ is the $s$-th entry of the similarity score matrix $S$ (say). The sixth column consists of weights of Covid class. Since Covid is a first class these weight are entries of first row of $wt_{m}$. The multiplication of the first row of the weight matrix $wt_{m}$ with $S$ gives logit for the Covid class. Similarly, logit for Normal and Pneumonia classes can be obtained by multiplying second and third row of the weight matrix $wt_{m}$ with the matrix $S$, respectively. Hence, the logits for the first, second and third classes are 0.752591, −0.627040 and −0.623544, respectively. The matrix $S$ and transpose of $wt_{m}$ that we obtain from our experiments are:

\begin{align*} \left [{ \begin{array}{c} 0.042843\\ 0.087834\\ 0.011572\\ 0.011572\\ 0.011572\\ 0.011572\\ 0.011572\\ 0.011572\\ 0.011572\\ 0.011572\\ 0.000106\\ 0.000104\\ 0.000108\\ 0.000106\\ 0.000094\\ 0.000108\\ 0.000106\\ 0.000104\\ 0.000104\\ 0.000110\\ 0.000077\\ 0.000084\\ 0.000078\\ 0.000082\\ 0.000085\\ 0.000085\\ 0.000057\\ 0.000085\\ 0.000085\\ 0.000082\\ \end{array}}\right], \left [{ \begin{array}{ccc}3.421200& -2.861400&-2.842300\\ 3.360900& -2.848000& -2.845600\\ 3.407200& -2.773600& -2.735800\\ 3.356100& -2.754100& -2.731300\\ 3.356100& -2.754100& -2.731300\\ 3.356100& -2.754100& -2.731300\\ 3.407200& -2.773600& -2.735800\\ 3.368500& -2.767400& -2.744300\\ 3.356100& -2.754100& -2.731300\\ 3.252000& -2.644300& -2.621000\\ -0.751730& 3.291000& -0.000065\\ -0.297610& 3.254100& -0.000003\\ -0.000024& 2.969000& 0.000019\\ -0.750210& 3.305100& -0.000021\\ -2.835900& 3.325100& -2.634000\\ -0.000024& 2.969000& 0.000019\\ -0.751730& 3.291000& -0.000065\\ -0.284340& 3.272900& -0.000005\\ -0.297610& 3.254100& -0.000003\\ 0.000015& 3.254800& 0.000001\\ -2.095800& -0.763840& 3.590500\\ -0.000033& -0.000012& 3.394200\\ -0.000008& 0.000015& 3.154800\\ -2.205300& -1.313500& 3.508200\\ -0.000449& -0.000025& 3.582100\\ -0.066042& -0.000073& 3.557700\\ -2.832500& -2.831200&3.335500\\ -0.000449& -0.000025& 3.582100\\ -0.000449& -0.000025& 3.582100\\ -2.272800& -1.308300& 3.562400\\ \end{array} }\right].\end{align*}

## VII. The Performance Description With Confusion Matrices

The comparison of the performance of Gen-ProtoPNet with NP-ProtoPNet, ProtoPNet and the base models is made with some metrics, such as: accuracy, precision, recall and F1-score. The confusion matrices are also used to outline the performance of Gen-ProtoPNet. A confusion matrix is an array that is used to describe the performance of a classification model on a set of test data for which the true values are known [54].

True positive (TP) is the number of items correctly labeled as belonging to the positive class, that is, the items are predicted to belong to a class when they actually belong to that class. True negative ( $TN$) is the number of items for which the model correctly predict the negative classes, that is, the items are predicted to not belonging to a class when they actually belong to other classes, see [55]. Note that, in non-binary classifications, $TP$ and $TN$ are the diagonal entries of the confusion matrix. False positive ( $FP$) is the number of items incorrectly predicted as belonging to the positive class. False negative ( $FN$) is the number of items incorrectly predicted as not belonging to the positive class, see [52]. The metrics accuracy, precision and recall in terms of the above positives and negatives are:

\begin{equation*} \dfrac {TP + TN}{\text {Total Cases}}, \dfrac {TP}{TP+FP} {~\text {and }} \dfrac {TP}{TP+FN}, {~\text {respectively}}.\end{equation*}

F1-score is the harmonic mean of precision and recall, that is, $\text {F1-score} = \dfrac {2}{{\text {Precision}^{-1}}+ {\text {Recall}^{-1}}}$, see [53].

**FIGURE 5.**
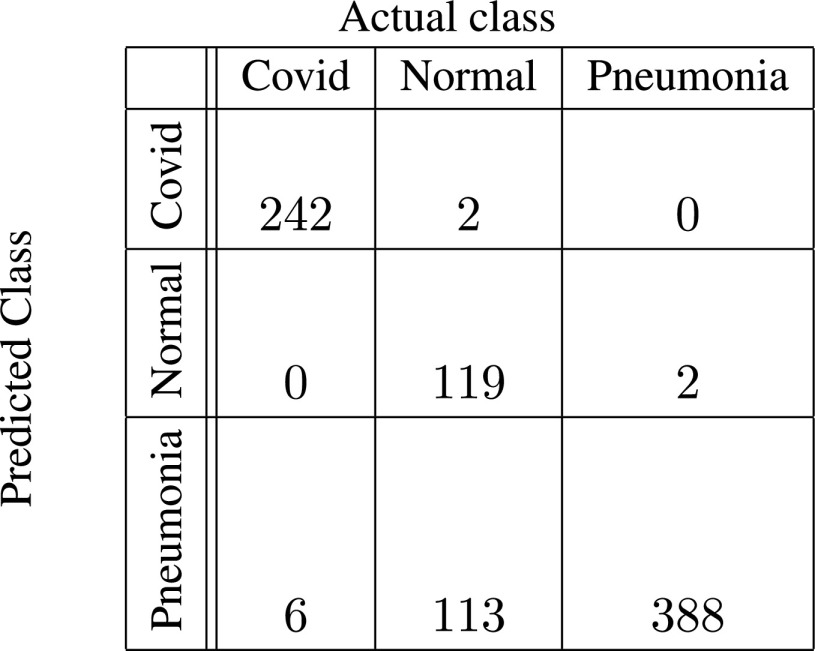
Gen-ProtoPNet with base VGG-16.

**FIGURE 6.**
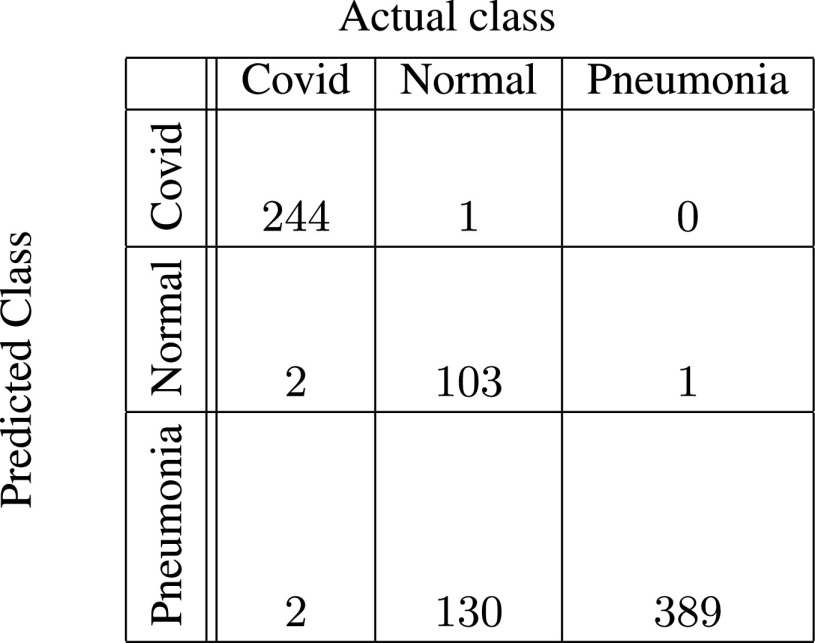
Gen-ProtoPNet with base VGG-19.

**FIGURE 7.**
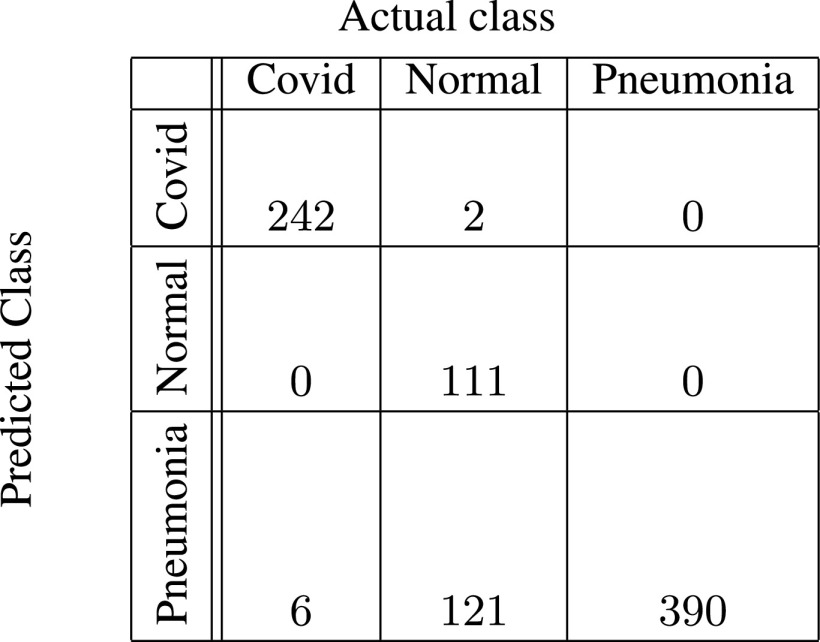
Gen-ProtoPNet with ResNet-34.

**FIGURE 8.**
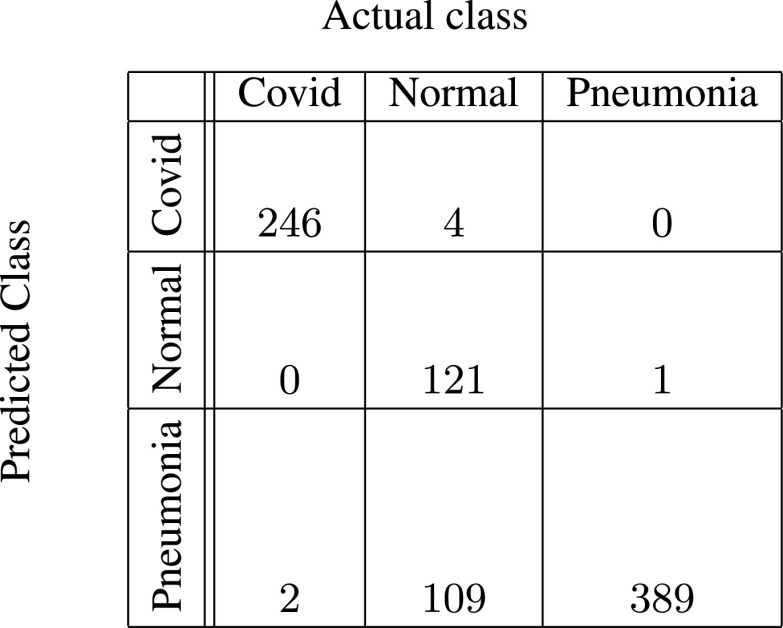
Gen-ProtoPNet with ResNet-152.

**FIGURE 9.**
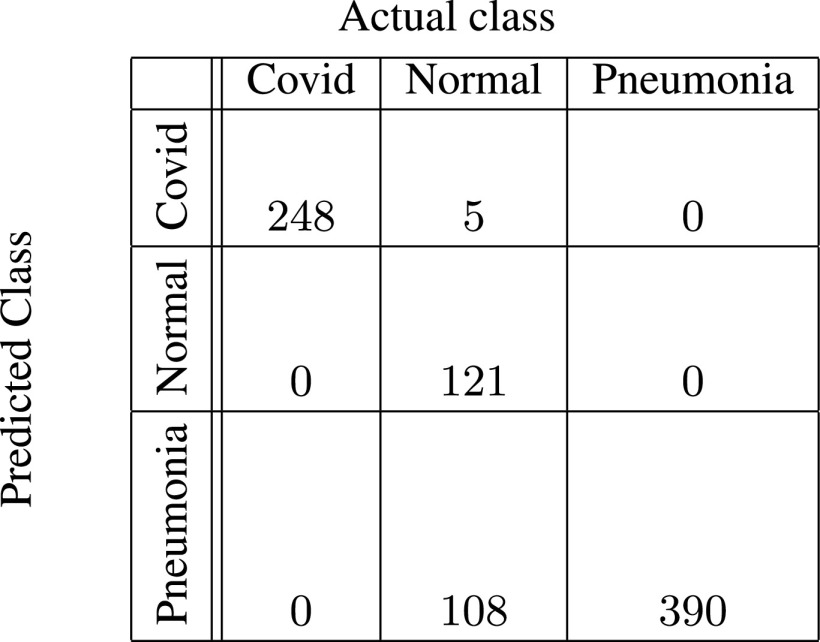
Gen-ProtoPNet with DenseNet-121.

**FIGURE 10.**
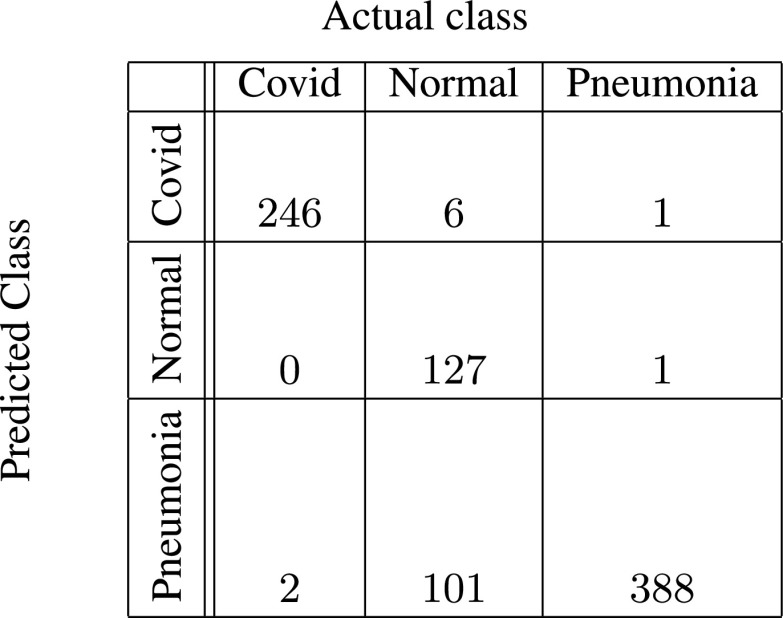
Gen-ProtoPNet with DenseNet-161.

In figures 5–10, the confusion matrices give visualization of the performance of Gen-ProtoPNet with the six baselines. Let $H$ be any of the following six confusion matrices. Suppose $(r, s)$ entry of the confusion matrix $H$ is given by $H[r][s]$. Therefore, $TP$ for the first class (Covid) are $H[{0}][{0}]$, and $TN$ are $H[{1}][{1}] + H[{2}][{2}]$. In addition, $FP$ and $FN$ for the first class are $H[{0}][{1}]+H[{0}][{2}]$ and $H[{1}][{0}]+H[{2}][{0}]$ respectively. Next, we describe the confusion matrix (given in Figure 5) for Gen-ProtoPNet when constructed over baseline VGG-16. Total correct predictions made by Gen-ProtoPNet with baseline VGG-16 are 749 (=242 + 119 + 388), see Figure 5. Total number of test images are 872, see Section V. Thus, the accuracy is 85.89%. The above definitions and Figure 5 give us the precision, recall and F1-score equal to 0.99, 0.98 and 0.98, respectively. Similarly, the metrics for Gen-ProtoPNet with the other baselines can be determined from Figures 6–10.

## VIII. Comparison of the Performance of Gen-ProtoPNet With the Performance of NP-ProtoPNet, ProtoPNet and the Baselines

The convolution layers of several neural networks can be used to build the models Gen-ProtoPNet, NP-ProtoPNet and ProtoPNet. As stated in Section V, we trained and tested Gen-ProtoPNet with the baseline models over the datesets of the $X$-rays [13], [24]. Also, NP-ProtoPNet and ProtoPNet were examined over the same datasets and with the same base models. We trained and tested all models that are compared in Table 1 for 500 epochs.

The measures of the performances of the models (Gen-ProtoPNet, NP-ProtoPNet and ProtoPNet with the six base models) in the metrics can be found in Table 1. Also, the measures of the performance the base models themselves are given in Table 1. We explain the Table 1 with an account of the performance of each of these models with base model VGG-16. However, the measures of the performance of these models with the other five base models are also given in Table 1.

In the second column of the Table 1, the spacial dimensions of prototypes corresponding to each base model are given. For example, when Gen-ProtoPNet is constructed over the base model VGG-16, and prototypes have spacial dimension $4\times 6$ then the accuracy, precision, recall and F1-score of Gen-ProtoPNet are 85.89, 0.99, 0.98 and 0.98, respectively. Similarly, the measures of the performances of the models NP-ProtoPNet and ProtoPNet with baseline model VGG-16 in the metrics accuracy, precision, recall and F1-score are 84.63, 0.97, 0.99, 0.97, and 79.93, 0.87, 0.92, 0.89, respectively. Also, the measures of the performances of VGG-16 itself (Base only) in the metrics accuracy, precision, recall and F1-score are 82.45, 0.97, 0.98 and 0.97, respectively.

The performance of Gen-ProtoPNet is improved over ProtoPNet with all the base models. Also, the performance of Gen-ProtoPNet is better than the performance of NP-ProtoPNet with some baseline models, and in two cases its performance is better than the performance of the baseline models themselves.

## IX. Graphical Comparison of the Accuracies of the Models

**FIGURE 11.**
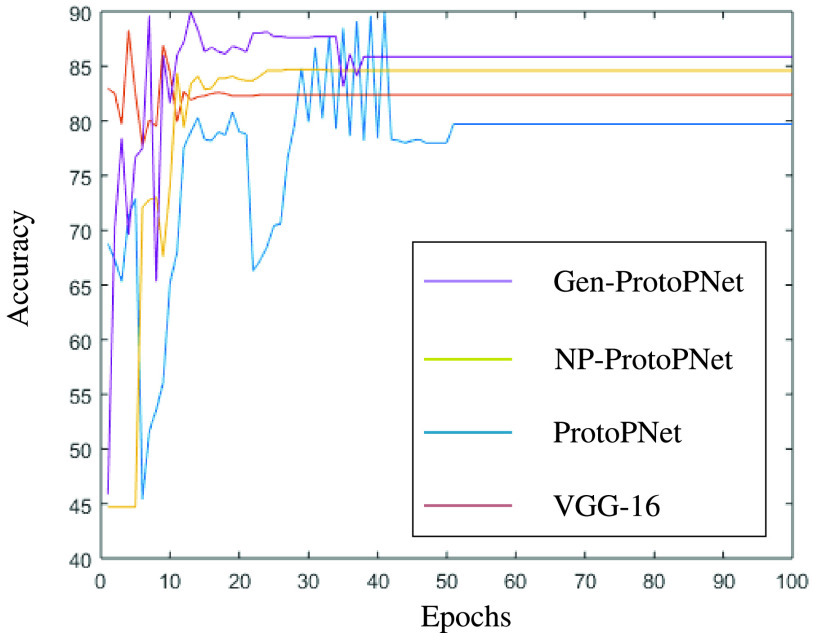
Accuracy comparison with baseline VGG-16.

**FIGURE 12.**
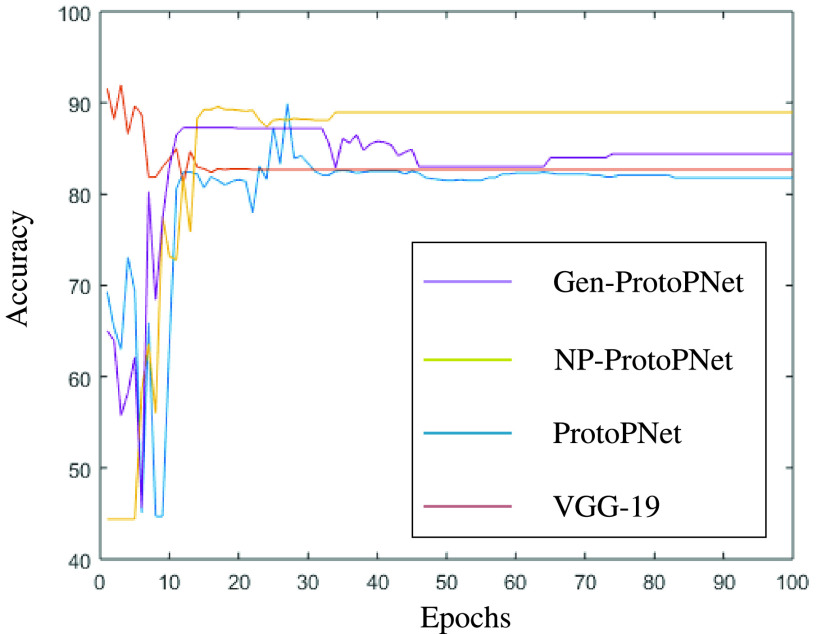
Accuracy comparison with baseline VGG-19.

**FIGURE 13.**
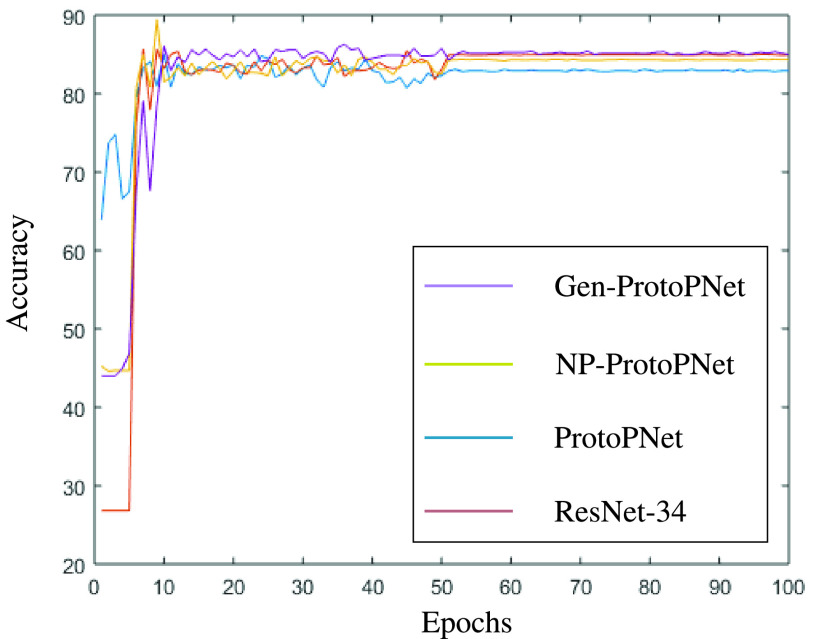
Accuracy comparison with baseline ResNet-34.

**FIGURE 14.**
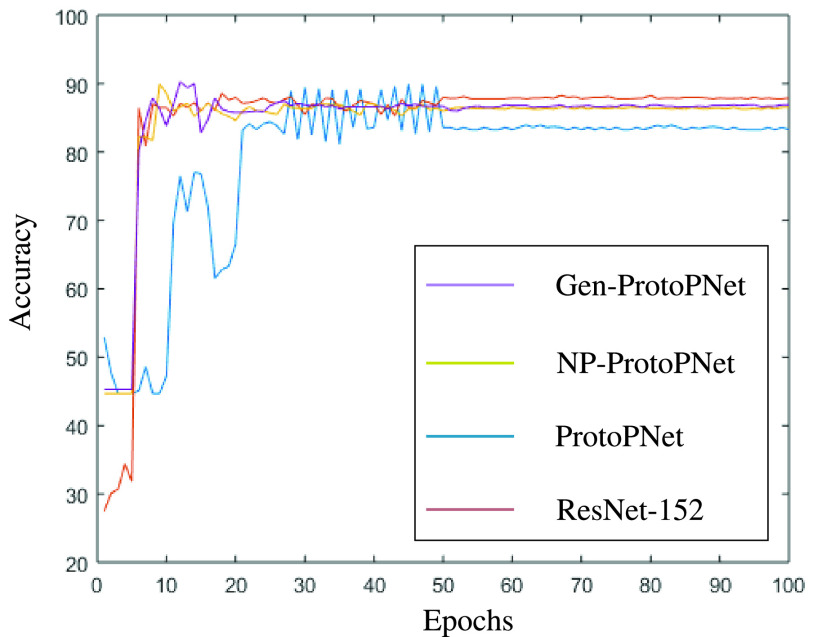
Accuracy comparison with baseline ResNet-152.

**FIGURE 15.**
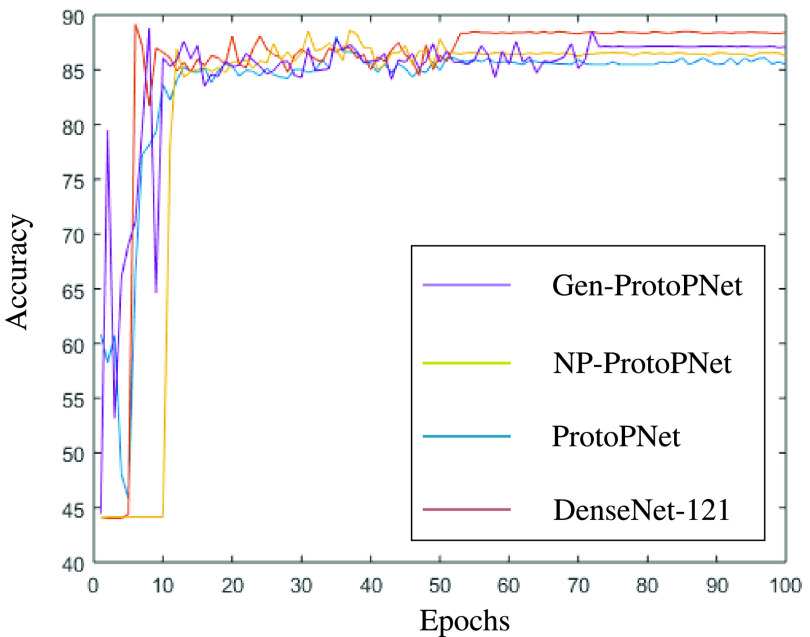
Accuracy comparison with baseline DenseNet-121.

**FIGURE 16.**
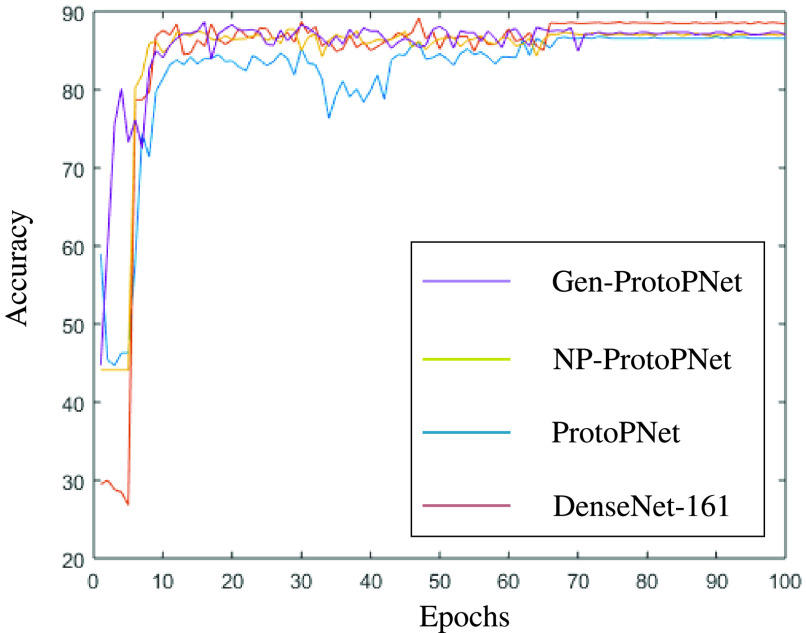
Accuracy comparison with baseline DenseNet-161.

In this section, a graphical comparison of the accuracies of Gen-ProtoPNet with the other models is provided over 100 epochs. In the figures 11–16, the curves of colors purple, yellow, blue and brown sketch the accuracies of Gen-ProtoPNet, NP-ProtoPNet, ProtoPNet and the baselines. For example, in Figure 11, the accuracies of Gen-ProtoPNet, NP-ProtoPNet and ProtoPNet with the base model VGG-16, and the base model VGG-16 itself are depicted.

The performance in accuracy of Gen-ProtoPNet is the highest with the baseline model VGG-16 and second highest for the remaining base models except VGG-19. Therefore, the curve depicting the accuracy for Gen-PrortoPNet is the highest for base model VGG-16 and second highest for the other base models.

The accuracies given by these models become stable before 100 epochs. Although, we experimented these models for 500 epochs, but the comparison of the accuracies of these models is outlined in the figures 11–16 only for first 100 epochs to make the shape of the curves more clearer in the beginning as accuracy of each of these models stabilizes before 100 epochs.

## X. Limitations

The convex optimization of the last layer of our model takes considerable time during the training of our model. Our experiments show that zero connection between similarity scores and incorrect classes is hard to achieve during the training process. However, this technique of convex optimization of the last layer is adopted from ProtoPNet model that has already been published in one of the top conferences [7].

## XI. Conclusion

Gen-ProtoPNet is closely related to two interpretable deep learning models ProtoPNet and NP-ProtoPNet that calculate the similarity scores between prototypes of spacial dimension $1\times 1$ and patches of an input image by finding the $L2$ distance between the prototypes and the patches. In our model, we use a generalized version of the distance function $L2$ that enables us to use prototypes of any type of spacial dimensions, that is, square spacial dimensions and rectangular spacial dimensions. The use of rectangular spacial dimensions of prototypes enabled our model to improve its performance over ProtoPNet model.
